# Na-rich layered Na_2_Ti_1−x_Cr_x_O_3−x/2_ (x = 0, 0.06): Na-ion battery cathode materials with high capacity and long cycle life

**DOI:** 10.1038/s41598-017-00346-x

**Published:** 2017-03-23

**Authors:** Shufeng Song, Masashi Kotobuki, Yingqian Chen, Sergei Manzhos, Chaohe Xu, Ning Hu, Li Lu

**Affiliations:** 1 College of Aerospace Engineering, Chongqing University, Chongqing, 400044 P.R. China; 2 Materials Science Group, Department of Mechanical Engineering, National University of Singapore, Singapore, 117575 Singapore; 3 The State Key Laboratory of Mechanical Transmissions, Chongqing University, Chongqing, 400044 P.R. China; 4 National University of Singapore Suzhou Research Institute, Suzhou, P.R. China

## Abstract

Rechargeable lithium batteries have been well-known and indispensable for portable electronic devices, and have the potential to be used in electric vehicles and smart grids. However, the growing concerns about the availability of lithium resources for large-scale applications have revived interest in sodium ion batteries. Of many obstacles to commercialization of Na-ion batteries, achieving simultaneously a large reversible capacity and good cycling capability of electrode materials remains a major challenge. Here, we report a new cathode material, Na-rich layered oxide Na_2_Ti_0.94_Cr_0.06_O_2.97_, that delivers high reversible capacity of 336 mAh g^−1^ at current density of 18.9 mA g^−1^ along with promising cycling capability of 74% capacity retention over 1000 cycles at current of 378 mA g^−1^. The high capacity is associated to the redox reaction of oxygen, which is confirmed here by a combined experimental and theoretical study. The present work therefore shows that materials beyond mainstream layered oxides and polyanion compounds should be considered as candidate high-performance cathodes for Na-ion batteries.

## Introduction

Lithium ion batteries have become an indispensable energy storage technology for mobile electronics since their commercialization, and have promise for electric vehicles as well as stationary grid applications. Nonetheless, concerns about the availability of lithium resource, which is not considered as an abundant element owing to its highly non-uniform spread within the crust of the Earth^1^, the significant increase of the price of lithium carbonate during the past decade, the emerging need for inexpensive stationary energy storage, all argue for research on alternatives to lithium ion batteries and arouse the research on sodium ion batteries^2^. In contrast to lithium, the sodium resource is evenly distributed in the Earth's crust and sodium is one of the most abundant elements with a very low material cost. In addition, sodium is the second-lightest alkali element after lithium. Moreover, sodium possesses a low redox potential (−2.71 V vs S.H.E.), and similar electrochemistry to lithium. In view of these considerations, sodium ion batteries are the ideal alternatives to lithium ion batteries, in particular, for the stationary energy storage applications^3^.

Inspired by known lithium cathode materials, extensive works on sodium cathodes have been focused on conventional two-dimensional layered oxides Na_x_MO_2_ (0 < × < 1, M: electrochemically-active transition metal) and three-dimensional polyanion compounds due to their ability to accommodate the large ionic radius and atomic weight of sodium^4^. Historically, the intercalation chemistry of layered sodium oxides was first investigated, which exhibited poor electrochemical performance^5^. The layered sodium oxides are generally composed of O3 and P2 phases depending on the coordination environment for sodium and the number of stacking transition-metal layers. O3-type *α*-NaFeO_2_ (ref. 6), NaCrO_2_ (ref. 7), NaNiO_2_ (ref. 8), etc. all proved to be electrochemically active, but delivered capacities of less 120 mAh g^−1^, corresponding to only ~0.5 Na or less cycled per formula unit. Yabuuchi *et al.* ^9^ reported P2-type Na_2/3_Fe_1/2_Mn_1/2_O_2_ that delivered an exceptionally high capacity of ~190 mAh g^−1^ with the Fe^3+^/Fe^4+^ redox, but its cyclability was insufficient, with the reversible capacity reduced from 190 mAh g^−1^ to 150 mAh g^−1^ after 30 cycles. Similarly, monoclinic-phase *α*-NaMnO_2_ (ref. 10), orthorhombic-phase *β*-NaMnO_2_ (ref. 11), P2-type Mg-doped Na_0.67_Mn_0.8_Mg_0.2_O_2_ (ref. 12) and so on exhibited large capacities of 150~190 mAh g^−1^, but inferior cycle life. Johnson *et al.* (ref. 13) designed a Li-substituted layered P2-O3 intergrowth Na_1__−__x_Li_x_Ni_0.5_Mn_0.5_O_2+d_, and Zhou *et al.* (ref. 14) reported a layered P2-O3 Na_0.66_Li_0.18_Mn_0.71_Ni_0.21_Co_0.08_O_2+δ_ composite, that delivered a high capacity as well as better cyclability; in particular, for Na_0.66_Li_0.18_Mn_0.71_Ni_0.21_Co_0.08_O_2+δ_, a good capacity retention of 75% is obtained after 150 cycles at a 0.5 C rate (ca. 113 mAh g^−1^ vs initial 150 mAh g^−1^). It is unsurprising that major efforts have been devoted to the research on layered sodium oxides Na_x_MO_2_ owing to the huge commercial success of layered lithium oxides. However, a long cycle life is one of the major obstacles, and capacities must be improved further. On the other hand, the polyanion compounds possess superior cycling performance due to a strong covalent three-dimensional framework. For example, the NASICON-type Na_3_V_2_(PO_4_)_3_ delivered a capacity retention of 68% after 2000 cycles at a 5 C rate, achieved by confining carbon-coated Na_3_V_2_(PO_4_)_3_ nanoparticles in ordered mesoporous carbon^15^. However, the polyanion compounds generally suffer from low reversible capacities, due to the limited active sodium content and large molecular weight thus low theoretical capacities. For example, the initial capacities of typical polyanion cathodes Na_3_V_2_(PO_4_)_3_^15^, maricite-type NaFePO_4_^16^, Na_3.32_Fe_2.34_(P_2_O_7_)_2_^17^, and so on, were ~114, 142, and 85 mAh g^−1^, respectively, corresponding to the respective theoretical capacities of 118, 155, and 118 mAh g^−1^. Therefore, it is urgent to explore cathode materials which combine high capacities (reversible and theoretical capacities) and long cycle life. Here, we report a new class of sodium cathodes, Na_2_TiO_3_ and Na_2_Ti_0.94_Cr_0.06_O_2.97_, combining a high capacity ~336 mAh g^−1^ and a long cycle life as well as low material cost.

## Results

### Characterization of Na_2_TiO_3_ and Na_2_Ti_0.94_Cr_0.06_O_2.97_

**Figure 1:**
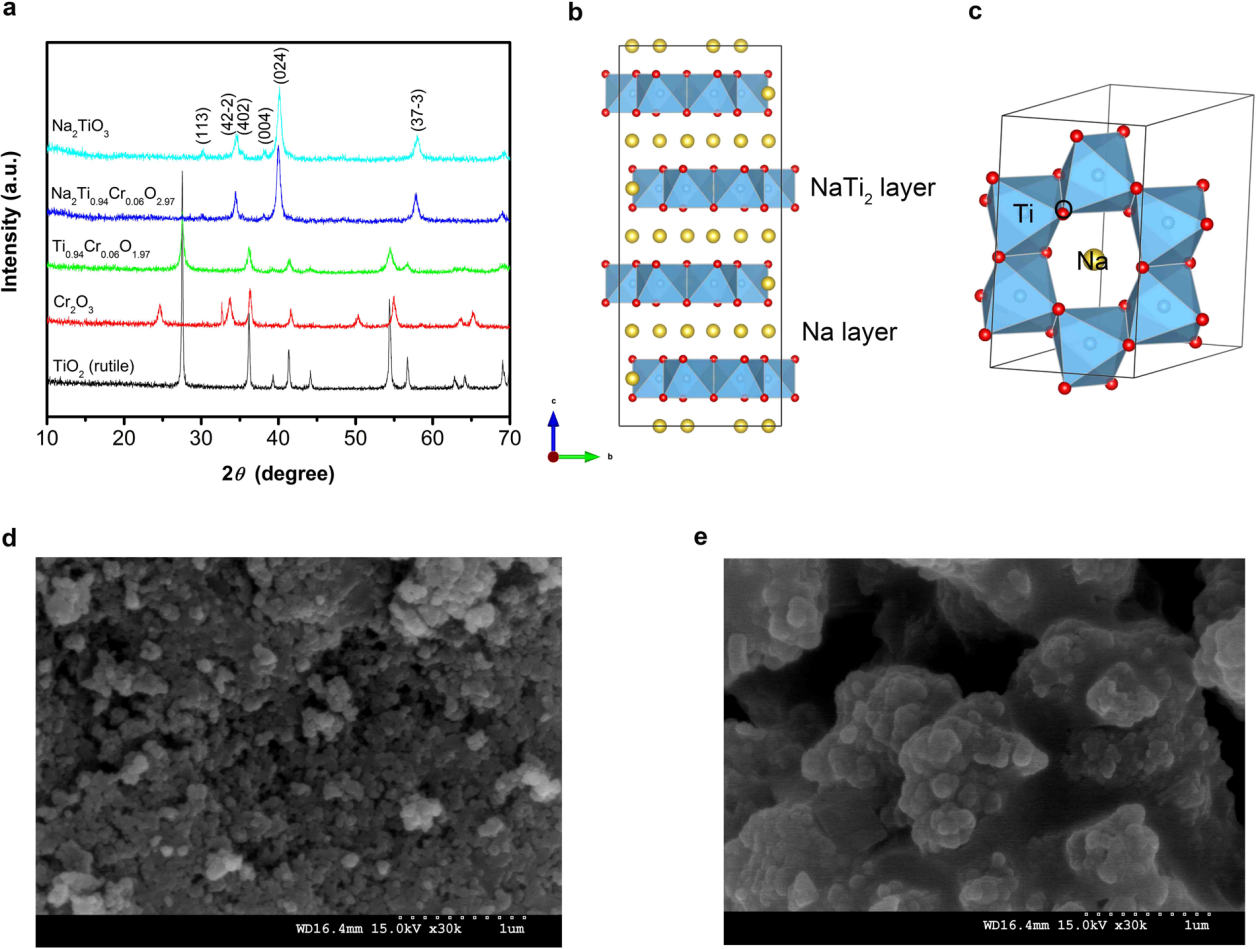
Material characterization of Na_2_TiO_3_ and Na_2_Ti_0.94_Cr_0.06_O_2.97_ synthesized at 500 °C for 6 h. (**a)** XRD patterns for raw materials TiO_2_, Cr_2_O_3_, TiO_2_-Cr_2_O_3_ solid solution, Na_2_TiO_3_ and Na_2_Ti_0.94_Cr_0.06_O_2.97_. (**b)** Crystal structure of Na_2_TiO_3_. (**c)** Polyhedral drawing of Na_1/3_Ti_2/3_ layers. SEM images of as-prepared Na_2_TiO_3_ (**d**) and Na_2_Ti_0.94_Cr_0.06_O_2.97_ (**e**).

Na_2_TiO_3_ and Na_2_Ti_0.94_Cr_0.06_O_2.97_ are synthesized *via* a solid-state reaction at 500 °C for 6 h in air. To prepare Na_2_Ti_0.94_Cr_0.06_O_2.97_, TiO_2_-Cr_2_O_3_ solid solution is first synthesized as precursor by high-energy ball-milling for 5 h. As shown in Fig. 1a, the TiO_2_-Cr_2_O_3_ solid solution exhibits reflections of rutile-phase TiO_2_, no Cr_2_O_3_ phase is present. Na_2_TiO_3_ has phases of *α*, *β* and *γ*, which exhibit face-centered cubic, monoclinic and monoclinic structures, respectively^18, 19^. As seen in Fig. 1a, Na_2_TiO_3_ and Na_2_Ti_0.94_Cr_0.06_O_2.97_ are single phase and are assigned to *β* phase and monoclinic structure with a space group *C* 2/*c*. The lattice parameters of Na_2_TiO_3_ are 13.021(0) Å, 13.922(0) Å and 9.526(0) Å. The lattice parameters of Na_2_Ti_0.94_Cr_0.06_O_2.97_ are 12.986(4) Å, 13.885(0) Å and 9.500(7) Å. It implies that small amount of addition of Cr does not change the lattice parameters significantly due to similar effective ionic radii of Ti^4+^ and Cr^3+^. Na_2_TiO_3_ can be reformulated as Na[Na_1/3_Ti_2/3_]O_2_ according to the Li-rich layered oxide Li_2_MnO_3_ (ref. 20). The monoclinic cells consist of alternating Na and Na_1/3_Ti_2/3_ layers stacked along the *c*-axis (Fig. 1b). Within Na_1/3_Ti_2/3_ layers, Na cations are surrounded by six Ti cations and form a hexagon (Fig. 1c). The size of particles of Na_2_TiO_3_ and Na_2_Ti_0.94_Cr_0.06_O_2.97_ is about 100 nm owing to the low as-prepared temperature (Fig. 1d,e).

### Electrochemical performances of Na_2_TiO_3_ and Na_2_Ti_0.94_Cr_0.06_O_2.97_

**Figure 2:**
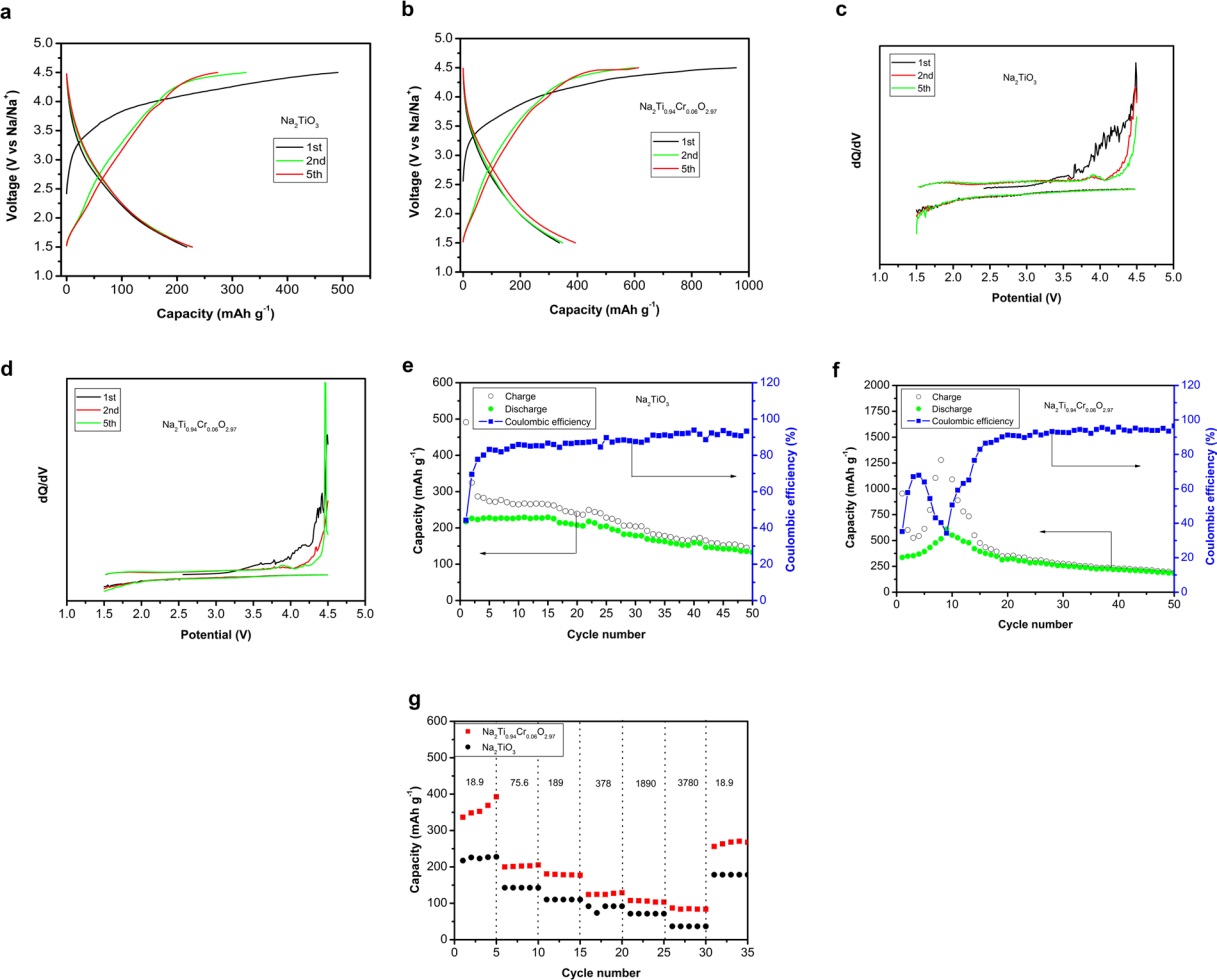
Electrochemical performances of Na_2_TiO_3_ and Na_2_Ti_0.94_Cr_0.06_O_2.97_. **(a,b)** Voltage profiles of Na_2_TiO_3_ and Na_2_Ti_0.94_Cr_0.06_O_2.97_. (**c,d)** dQ/dV plots of Na_2_TiO_3_ and Na_2_Ti_0.94_Cr_0.06_O_2.97_. (**e,f)** Cycling performance with Coulombic efficiency of Na_2_TiO_3_ and Na_2_Ti_0.94_Cr_0.06_O_2.97_ at current density of 18.9 mA g^−1^. (**g)** Rate capabilities of Na_2_TiO_3_ and Na_2_Ti_0.94_Cr_0.06_O_2.97_ at current density from18.9 mA g^−1^ to 3780 mA g^−1^.

Compared with the conventional layered sodium cathodes, the present electrode materials have several orders of magnitude lower electrical conductivities due to a lack of a transition-metal with multiple oxidation states. The low conductivities are overcome by making Na_2_TiO_3_/Na_2_Ti_0.94_Cr_0.06_O_2.97_ and Super P composites via simple high energy ball milling for 5 h. We believe that the extensive research experience of carbon coating of LiFePO_4_ can be used with the present materials^21^, thus the total carbon content can be significantly decreased, and the electrode loading and energy density can be further improved. The electrochemical performances of Na_2_TiO_3_ and Na_2_Ti_0.94_Cr_0.06_O_2.97_ are compared as shown in Fig. 2. One interesting phenomenon is that a long potential plateau can be observed at the end of first sodium extraction in voltage profile (Fig. 2a,b), which is a common feature for all the Li-rich layered cathodes^22^. Na_2_TiO_3_ delivers an initial discharge capacity of ~217 mAh g^−1^ in the voltage window of 1.5–4.5 V (versus Na/Na^+^) at a current density of 18.9 mA g^−1^ (Fig. 2a). Na_2_Ti_0.94_Cr_0.06_O_2.97_ delivers a much higher initial capacity of ~336 mAh g^−1^ (Fig. 2b).

The dQ/dV plots of 1^st^, 2^nd^ and 5^th^ cycles for both Na_2_TiO_3_ and Na_2_Ti_0.94_Cr_0.06_O_2.97_ are plotted in Fig. 2c,d. Both samples are similar. There are no pronounced peaks on dQ/dV plots, implying no distinct charge-discharge plateau. The reasons are due possibly to the electrochemical inactivity of Ti^4+^ and sluggish Na-ion diffusion in Na_2_TiO_3_ and Na_2_Ti_0.94_Cr_0.06_O_2.97_.

Another interesting phenomenon is that the activation processes are observed for both Na_2_TiO_3_ and Na_2_Ti_0.94_Cr_0.06_O_2.97_, especially for Na_2_Ti_0.94_Cr_0.06_O_2.97_, in which the discharge capacity is raised to a maximum value over initial several cycles (Fig. 2e,f). As seen in Fig. 2e, the discharge capacity of Na_2_TiO_3_ increases mildly from ~217 mAh g^−1^ to 229 mAh g^−1^ over initial 15 cycles. The initial Coulombic efficiency of Na_2_TiO_3_ is only ~44% and raises to 80% after initial 4 cycles. As seen in Fig. 2f, the discharge capacity of Na_2_Ti_0.94_Cr_0.06_O_2.97_ is much higher than that of Na_2_TiO_3_. The activation process is much more pronounced in that the capacity of Na_2_Ti_0.94_Cr_0.06_O_2.97_ raises from ~336 mAh g^−1^ to ~609 mAh g^−1^ over initial 9 galvanostatic cycles; after that, the capacity decays mildly to ~182 mAh g^−1^ after 50 galvanostatic cycles. The initial Coulombic efficiency of Na_2_Ti_0.94_Cr_0.06_O_2.97_ is ~35% and raises to 80% after initial 15 cycles. The increase in capacities over initial cycles can be attributed to the activation of the Na_2_Ti_0.94_Cr_0.06_O_2.97_, which is possibly related with sodium insertion to the TiO_2_ host structure. The decrease in capacities after full activation of Na_2_Ti_0.94_Cr_0.06_O_2.97_ is possibly attributed to the irreversible reaction of oxygen^23^.

The rate capabilities of Na_2_TiO_3_ and Na_2_Ti_0.94_Cr_0.06_O_2.97_ are shown in Fig. 2g, the Na_2_Ti_0.94_Cr_0.06_O_2.97_ exhibits higher capacities compared with Na_2_TiO_3_ at different current densities. Both the materials present good capacity retention. Particularly, the capacities of Na_2_TiO_3_ and Na_2_Ti_0.94_Cr_0.06_O_2.97_ can be recovered to ~178 and 268 mAh g^−1^, ca. 82% and 80% of the initial capacities at current density of 18.9 mA g^−1^, respectively.

### Electrical conductivities of Na_2_TiO_3_ and Na_2_Ti_0.94_Cr_0.06_O_2.97_

**Figure 3:**
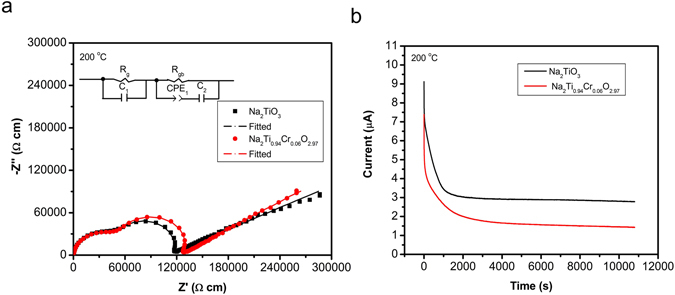
Conductivities of Na_2_TiO_3_ and Na_2_Ti_0.94_Cr_0.06_O_2.97_ measured in air at 200 °C. (**a)** AC impedance plots •◾: Experimental values. The solid line represents simulated data using an equivalent circuit of (R_g_C_1_)(R_gb_CPE_1_C_2_) (where R_g_ and R_gb_ are the resistances of grain and grain-boundary respectively, CPE is the constant phase element, C is the capacitor). (**b)** DC polarization measurement at constant voltage of 1 V using Ag blocking electrodes.

The electrical conductivities of Na_2_TiO_3_ and Na_2_Ti_0.94_Cr_0.06_O_2.97_ are evaluated by measurements of AC impedance and DC polarization at 200 °C (Fig. 3). The AC impedance plots (Fig. 3a) are simulated with an equivalent circuit of (R_g_C_1_)(R_gb_CPE_1_C_2_), where R_g_, R_gb_, C and CPE mean bulk resistance, grain-boundary resistance, capacitor element, and constant phase element, respectively. The bulk conductivity and boundary conductivity are calculated based on the two well-separated semicircles. The bulk conductivity of Na_2_Ti_0.94_Cr_0.06_O_2.97_ (2.0 × 10^−5^ S cm^−1^) is slightly larger than that of Na_2_TiO_3_ (1.7 × 10^−5^ S cm^−1^). The bulk conductivities of Na_2_Ti_0.94_Cr_0.06_O_2.97_ and Na_2_TiO_3_ are much higher than that of intrinsic conductivity of LiFePO_4_ (~10^−9^ S cm^−1^)^24^, which implies that there is much space to improve the performance of the present materials if advanced carbon coating process is developed. The diffusion of Na cations in bulk grains would determine the redox processes in the case of active material-carbon composites. The possible creation of oxygen vacancies in Na_2_Ti_0.94_Cr_0.06_O_2.97_ would weaken the binding of oxygen anions to migrating Na cations, thus favoring the migration of sodium ions in bulk and leading to higher bulk conductivity and better sodium deintercalation/intercalation performance. The electron conductivities are evaluated by DC polarization at 200 °C and with a constant voltage of 1 V using Ag blocking electrodes (Fig. 3b). The electron conductivities of Na_2_TiO_3_ and Na_2_Ti_0.94_Cr_0.06_O_2.97_ are calculated to be 1.7 × 10^−6^ S cm^−1^ and 0.97 × 10^−6^ S cm^−1^ based on the used voltage and steady-state current, respectively. The transference numbers of sodium ions of Na_2_TiO_3_ and Na_2_Ti_0.94_Cr_0.06_O_2.97_ are calculated to be ~0.81 and 0.88, respectively, by means of (total conductivity-electron conductivity)/total conductivity, which demonstrates that the present materials are mixed ions and electrons conductive; this is advantageous for electrode materials.

### Cycling capability of Na_2_Ti_0.94_Cr_0.06_O_2.97_

**Figure 4:**
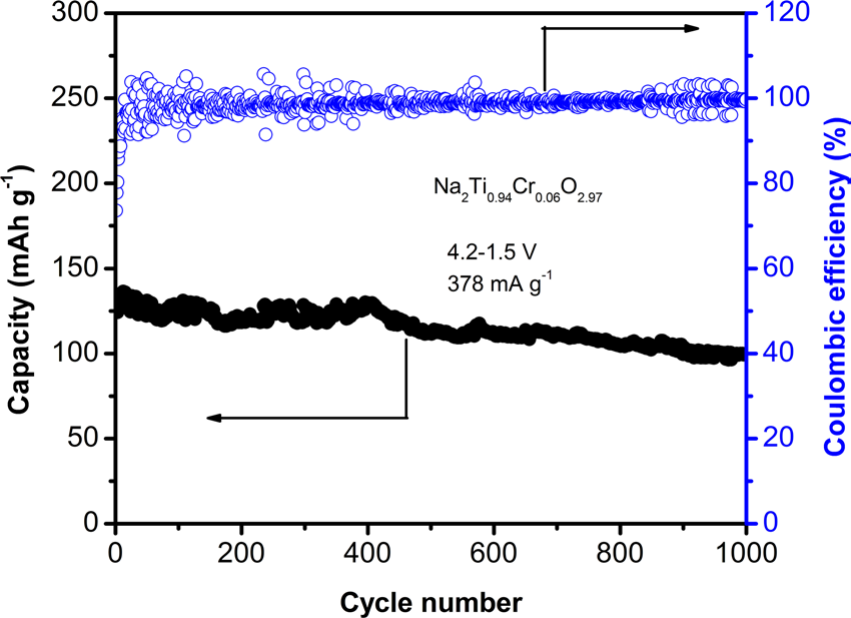
Long-term cycling of Na_2_Ti_0.94_Cr_0.06_O_2.97_ at current density of 378 mA g^−1^.

The long-term cycling of Na_2_Ti_0.94_Cr_0.06_O_2.97_ is performed at current density of 378 mA g^−1^ as shown in Fig. 4. The initial discharge capacity is ~124 mAh g^−1^, and raises to ~136 mAh g^−1^ after 15 cycles. The initial Coulombic efficiency is 74%, and is above 90% after initial 10 cycles. The reversible capacity is still 100 mAh g^−1^ after 1000 cycles, delivering a very low capacity decay rate of 0.026% per cycle, and a capacity retention of 74%, which demonstrates promising cycling capability of the material. It is noted that the present materials exhibit capacity decay with a charging voltage of 4.5 V, but good long-term cycling capability as the charging voltage decreases to 4.2 V. This implies that the capacity decay is related to the high voltage which is observed by another Na-rich layered oxide Na_2_Ru_0.75_Sn_0.25_O_3_ (ref. 25).

### Raman spectroscopy

**Figure 5:**
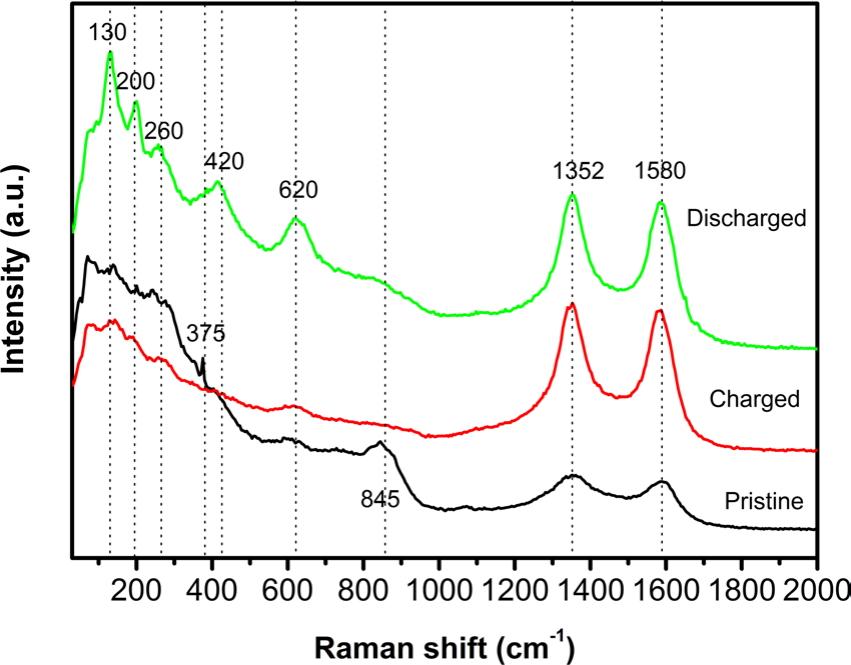
Raman spectra of pristine, charged and discharged Na_2_Ti_0.94_Cr_0.06_O_2.97_.

Raman spectroscopy has been employed to evaluate the structural evolution of Na_2_Ti_0.94_Cr_0.06_O_2.97_ upon desodiation and sodiation. The obtained Raman spectra are shown in Fig. 5. Bands at 1352 and 1580 cm^−1^ correspond to the D and G modes of Super P^26^. Thanks to Bamberger and Begun's pioneer work on Raman spectra for *α*-, *β*- and *γ*-Na_2_TiO_3_ (ref. 19), we can characterize the structural evolution of Na_2_Ti_0.94_Cr_0.06_O_2.97_ using Raman spectra. Two peaks around 845 cm^−1^ and one sharp peak at 375 cm^−1^ for the pristine sample can be characterized to *β* phase. Two distinct peaks at 260 and 130 cm^−1^ for the discharged sample also can be characterized to *β* phase, while three pronounced Raman peaks at 620, 420 and 200 cm^−1^ for the discharged sample can be characterized to *γ* phase. Therefore, it is indicated that ball-milled Na_2_Ti_0.94_Cr_0.06_O_2.97_ is mainly *β* phase and with minor *γ* phase, while *γ* phase is the major phase and *β* phase is the minor phase after discharge. The Raman spectrum of charged Na_2_Ti_0.94_Cr_0.06_O_2.97_ is poorly resolved and broad, implying that the charging process leads to certain degree of amorphization and disorder which is in agreement with the Density Functional Calculations. On the other hand, the discharged sample shows sharp Raman peaks compared with those of pristine and charged samples, indicating that better crystallization of monoclinic phase.

### XPS

**Figure 6:**
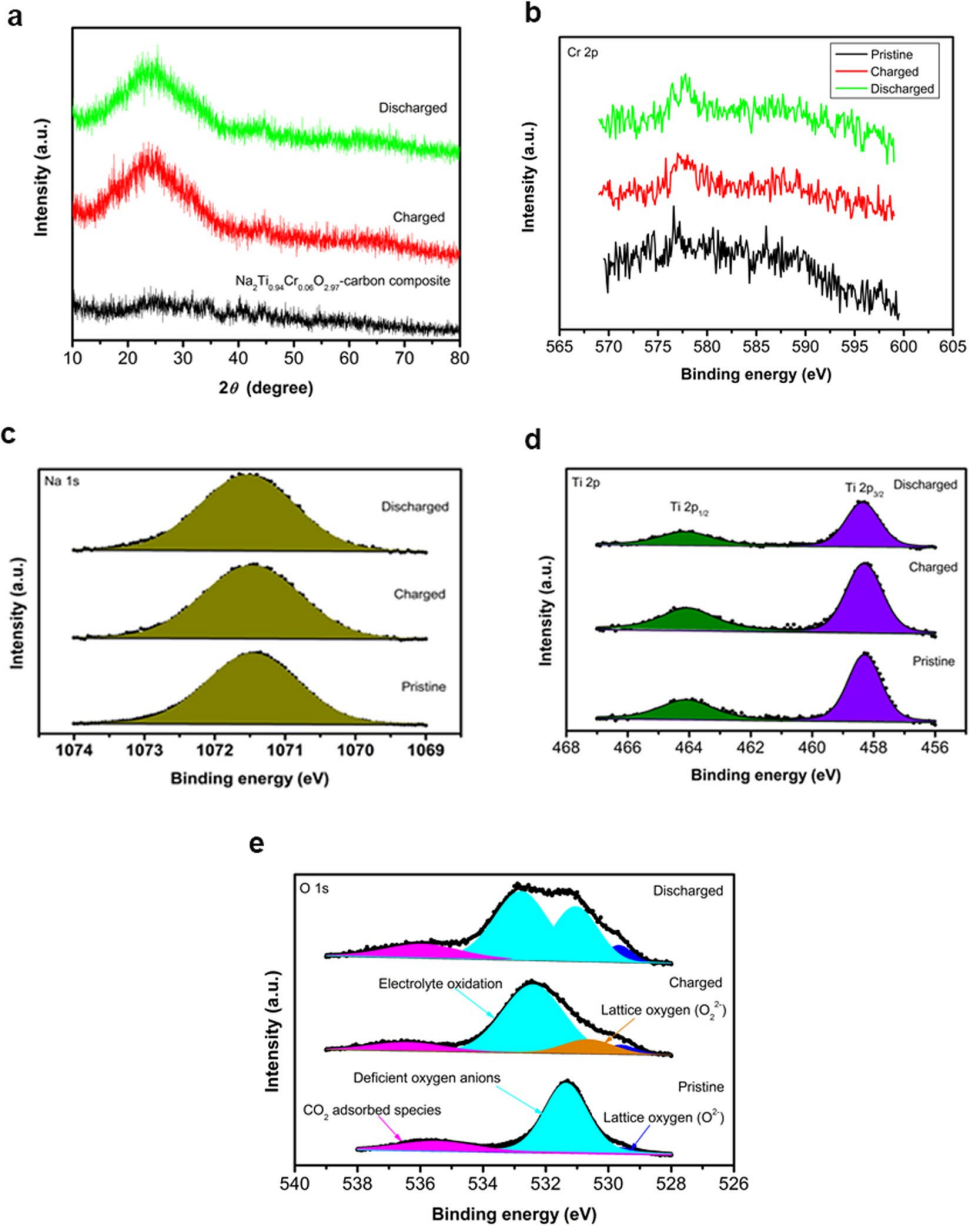
Analysis of the electrochemical desodiation and sodiation of Na_2_Ti_0.94_Cr_0.06_O_2.97_. **(a)** XRD patterns for the Na_2_Ti_0.94_Cr_0.06_O_2.97_-carbon composite, pristine electrode, fully charged sample, and discharged to 1.5 V under a current density of 18.9 mA g^−1^. XPS spectra of the Na_2_Ti_0.94_Cr_0.06_O_2.97_ electrodes, the spectra collected for pristine sample, fully charged sample and discharged to 1.5 V as well as by least-squares-fits using a software of XPSPEAK41. (**b)** Cr 2p spectra. (**c)** Na 1 s spectra. (**d)** Ti 2p spectra. **(e)** O 1 s spectra.

As shown in Fig. 6a, the X-ray diffraction peaks for Na_2_Ti_0.94_Cr_0.06_O_2.97_-carbon composite are very weak after high energy ball milling for 5 h. The composite electrodes are fully charged, which corresponds to extraction of two moles of Na, and subsequently discharged to 1.5 V. The charged and discharged samples display diffraction peak around 44 degree, which can be attributed to monoclinic *γ* phase. XPS spectra are applied to examine the change in the surface of the Na_2_Ti_0.94_Cr_0.06_O_2.97_ electrode during desodiation/sodiation. The Cr 2p peaks are around 577 eV which can be assigned to Cr^3+^ component, though the Cr 2p spectra are poorly resolved (Fig. 6b). Figure 6c shows the Na 1 s spectrum. The signal is stable at ~1071.5 eV upon desodiation/sodiation, indicating the valence state of sodium cations (Na^1+^) is not affected during desodiation/sodiation process^27^. Two strong signals at ~458.3 eV and 464.1 eV for the present material are ascribed to Ti 2p_3/2_ and Ti 2p_1/2_, respectively (Fig. 6d), corresponding to Ti^4+^ according to the ref. 28. No other Ti chemical states are detected, indicating Ti^4+^ is electrochemical inactive in the present material. Huang *et al.* also reported Ti^4+^ was electrochemical inactive^29^. For the O 1 s spectrum of pristine Na_2_Ti_0.94_Cr_0.06_O_2.97_ (Fig. 6e), three main components are detected, one is an oxide ion (O^2−^) in the crystal lattice at 529.6 eV, the peak at 535.5 eV assigns the adsorbed species^22, 30^. The third peak at 531.4 eV may be assigned to NaOH and/or Na_2_O_2_, and is more probably due to oxygen anions of Na_2_Ti_0.94_Cr_0.06_O_2.97_ in the subsurface (bulk structure near the surface), which have a deficient coordination number^30–32^. The spectrum mildly changes when the sample is fully charged, the component at 532.4 eV is C = O bonds in polycarbonates, implying the decomposition of electrolyte solvents^33, 34^. The decomposition of polycarbonates is irreversible upon discharging, where the peak around 532–533 eV is remained. A new component at 530.6 eV corresponds to oxide ions with lower electronic density compared with O^2−^, namely O_2_^2−^ species^30, 35^. It is suggested that the lattice oxygen O^2−^ transforms partially to O_2_^2−^ upon the desodiation process. After the sodiation, the O_2_^2−^ component disappears, while the defective oxygen component partly remains. These phenomena demonstrate that the redox reactivity of oxygen is involved in the electrochemical desodiation and sodiation processes. We can quasi-quantificationally analyze the Na/Ti ratio from the XPS using the peak area and relative sensitivity factor (RSF) of element. Na/Ti ratio = [Area (Na)/RSF(Na)]/[Area (Ti)/RSF(Ti)]. It implies probably Na extraction upon charging and returning upon discharging (Table S1).

### Density Functional Calculations

**Figure 7:**
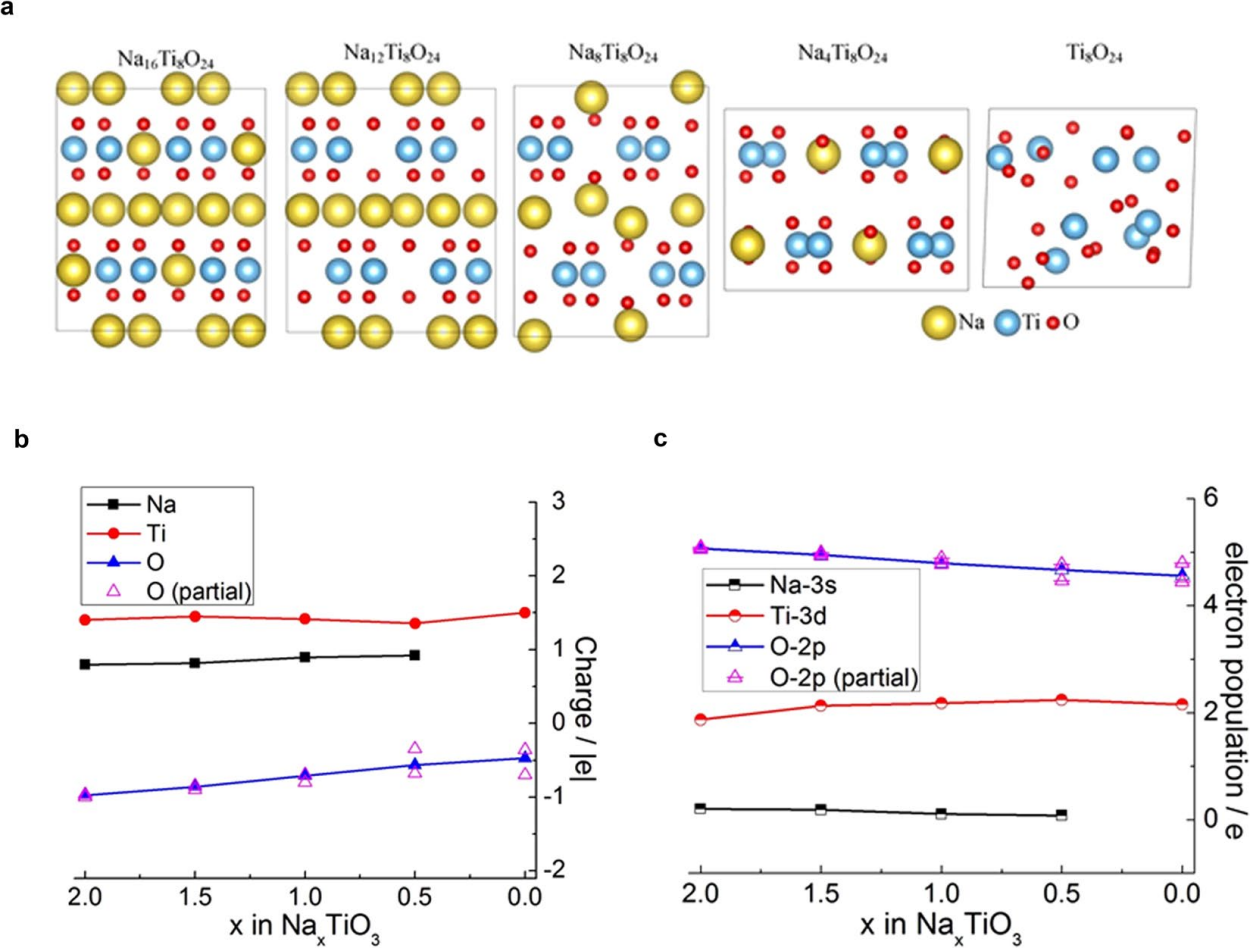
Density Functional Calculations for Na_2_TiO_3_. **(a)** Optimized structures of Na_16_Ti_8_O_24_, Na_12_Ti_8_O_24_, Na_8_Ti_8_O_24_, Na_4_Ti_8_O_24_ and Ti_8_O_24_ from left to right. (**b)** Mulliken and (**c)** Electron populations of the valence orbital of each element in Na_16_Ti_8_O_24_, Na_12_Ti_8_O_24_, Na_8_Ti_8_O_24_, Na_4_Ti_8_O_24_ and Ti_8_O_24_ which corresponds x = 2, 1.5, 1, 0.5, 0 in Na_x_TiO_3_ respectively. The lines connect average populations of each orbitals in different concentrations. The magenta triangles show average populations of 2p orbitals of different types of oxygen atoms at each concentration.

The participation of the oxygen redox is also confirmed by ab initio calculations. The computed crystalline structures of Na_16_Ti_8_O_24_, Na_12_Ti_8_O_24_, Na_8_Ti_8_O_24_, Na_4_Ti_8_O_24_ and Ti_8_O_24_ are shown in Fig. 7a. With the number of Na atoms in the cell decreasing, the structure eventually becomes disordered which corresponds to the instability of TiO_3_.

Mulliken charges of each element in Na_16_Ti_8_O_24_, Na_12_Ti_8_O_24_, Na_8_Ti_8_O_24_, Na_4_Ti_8_O_24_ and Ti_8_O_24_ are plotted in Fig. 7b. The Na atoms are ionized. With the increase of number of Na atoms in the cell, the charge of O increases (becomes more negative) while the charge of Ti is relatively stable. This indicates charge transfer from Na to O. There are two types of O atoms in this material: one is coordinated to Na and the other is coordinated only to Ti which have different Mulliken populations as shown by empty triangles in Fig. 7b. The full sodiation corresponds to the same charge balance as in the stoichiometric TiO_2_ with all oxygens in the O^2−^ states and the triangles in Fig. 7b coincide. As the material is desodiated, the charge difference between the two types of O increases. This is due to the valence electrons of Na occupying the 2p orbitals of O atoms which are coordinated to Na. During desodiation, the population of the 2p orbitals of these O atoms decreases, as the electrons leave the material together with Na ions, and the overall negative charge of these oxygen atom decreases as can be seen in Fig. 7c. We note that the absolute magnitudes of the charges are not equal to the assumed redox states (Ti^4+^, O^2−^) due to the use of Mulliken analysis with localized basis functions, as expected.

## Discussion

Among layered sodium oxides, P2-type Na_2/3_Fe_1/2_Mn_1/2_O_2_ (ref. 9), *α*-NaMnO_2_ (ref. 10), *β*-NaMnO_2_ (ref. 11), P2-type Na_0.67_Mn_0.8_Mg_0.2_O_2_ (ref. 12), and so on had been reported to deliver high discharge capacities of 150~190 mAh g^−1^, with the theoretical capacities of 264 mAh g^−1^, 244 mAh g^−1^, and 227 mAh g^−1^, respectively. Among typical polyanion compounds, NASICON-type Na_3_V_2_(PO_4_)_3_ (ref. 15), maricite-type NaFePO_4_ (ref. 16), Na_3.12_Fe_2.44_(P_2_O_7_)_2_ (ref. 17), and so on had reversible capacities of 114 mAh g^−1^, 142 mAh g^−1^ and 85 mAh g^−1^, with the theoretical capacities of 118 mAh g^−1^, 155 mAh g^−1^, and 118 mAh g^−1^, repectively. To the best of our knowledge, the present material benchmarks the highest-ever capacity by far discovered among other known sodium cathode materials. The high capacities are possibly related with the redox reaction of oxygen as demonstrated by XPS and Density Functional Calculations.

Besides high reversible capacities, the present Na_2_Ti_0.94_Cr_0.06_O_2.97_ exhibits promising cycling capability with capacity retention of 74% after 1000 cycles. The conventional layered sodium oxides have limited cycling capability because of a possibility of multiple phase transitions during cycling, i.e. a structural instability^36^, which is facilitated by a large ionic radius of Na. The as-prepared Na_2_Ti_0.94_Cr_0.06_O_2.97_ possess *β* phase and monoclinic Na-rich layered structure, which is transformed to mixed phases of *β* and *γ* upon discharging. Both *β* and *γ* phases possess monoclinic structures, which contributing the promising cycling capability.

In summary, we for the first time have investigated Na-rich layered compound Na_2_TiO_3_ as a new cathode for the Na-ion batteries. We find that Na_2_TiO_3_ cathode has a high reversible capacity of ~217 mAh g^−1^. Carbon coating had to be used to overcome its low conductivity. By doping Na_2_TiO_3_ with Cr^3+^ to create oxygen vacancies and to facilitate the migration of Na-ions, we achieve a much higher reversible capacity of ~336 mAh g^−1^. Furthermore, we demonstrate that the new Na-rich layered cathode Cr-doped Na_2_TiO_3_ shows long-term cycling capability (74% capacity retention after 1000 cycles, a very low capacity decay rate of 0.026% per cycle), which is superior to most mainstream layered sodium cathodes. A challenge for future work is to reduce the concentration of carbon and to improve the conductivity. We strongly believe that the present work will not only open up a viable strategy for designing and discovering new sodium cathodes, but also inspire future success in exploring superior electrode materials for next-generation rechargeable batteries.

## Methods

### Materials synthesis

To synthesize the Na_2_TiO_3_, proper amounts of TiO_2_ (Sigma-Aldrich, 99.9%) and NaOH (Sigma-Aldrich, 10% excess) were milled for 4 h. The mixture was then heated in alumina crucibles at 500 °C for 6 h under atmosphere of air. To synthesize Na_2_Ti_0.94_Cr_0.06_O_2.97_, TiO_2_-Cr_2_O_3_ solid solution was first synthesized by mixing TiO_2_ and Cr_2_O_3_ (Sigma-Aldrich) for at least 24 h followed by high-energy ball-milling for 5 h (SPEX SamplePrep 8000 M Mixer), then homogenized with 10% excess of NaOH and annealed at 500 °C for 6 h under air atmosphere. To prepare the electrode-carbon composite, Na_2_TiO_3_ or Na_2_Ti_0.94_Cr_0.06_O_2.97_ was mixed with Super P (TIMCAL Ltd.) in a weight ratio of 1: 1 and was milling under Ar atmosphere for 5 h (SPEX SamplePrep 8000 M Mixer).

### XRD and SEM

The XRD measurements were performed in a 2*θ* range of 10–70° using Shimadzu XRD-6000 Cu-Kα. The microstructure of powdered materials was examined using SEM (S-4300 Shimadzu).

### Conductivity

The bulk conductivity measurements were evaluated by AC impedance spectroscopy (Solartron 1260/1287) at 200 °C, with applying potential of 10 mV from 32 MHz to 1 Hz. The Na_2_TiO_3_ and Na_2_Ti_0.94_Cr_0.06_O_2.97_ powders were pelletized and sintered at 550 °C for 6 h under air atmosphere. Conductive Ag paste was coated on two sides of the pellets to form ion-blocking electrodes. To perform DC measurements, conductive Ag paint (>1000 S cm^−1^) was coated on two sides of the Na_2_TiO_3_ and Na_2_Ti_0.94_Cr_0.06_O_2.97_ pellets, and dried at 120 °C for 5 min. A constant voltage of 1 V is applied after the coated pellets were held for 1 h at 200 °C under air atmosphere, and variation of DC current was monitored until steady-state current was obtained.

### Electrochemical characterization

The battery performance was tested using 2016-type coin cell on MACCOR and LAND battery cycler instruments. The working electrode was prepared by mixing 80 wt% Na_2_TiO_3_/Na_2_Ti_0.94_Cr_0.06_O_2.97_-carbon active material, 10 wt% polyvinylidene fluoride (PVDF, Sigma), and 10 wt% Super P (TIMCAL Ltd.) in N-methylpyrrolildone (NMP, Sigma) solvent for at least 12 h. The electrode slurry was coated on aluminum foils and dried at 120 °C in vacuum for at least 12 h, with an average active material loading of ~0.6 mg cm^−2^. The electrolyte is 1 M NaClO_4_ in ethylene carbonate (EC)/propylene carbonate (PC) (1:1 in weight). The glass microfiber filter (Whatman, GF/A) was dried at 50 °C in vacuum for 48 h and was used as separator. The 2016-type coin cell was assembled with working electrode, electrolyte, separator, and metallic sodium (Sigma, 99.99%) in Ar-filled glove box. The galvanostatic charge and discharge measurements were conducted in the voltage window of 4.5–1.5 V and 4.2–1.5 V at different current densities from18.9 mA g^−1^ to 3780 mA g^−1^.

### Raman

Raman spectra were recorded with Horiba Jobin Yvon Modular Raman Spectrometer using 514 nm Stellar Pro Argon-ion laser. The system was calibrated using a silicon reference before the measurement (520.5 cm^−1^). The mixture of Na_2_Ti_0.94_Cr_0.06_O_2.97_ and Super P with ball milling 5 h is as pristine sample. For the first-cycled charged and discharged samples, the cells were fully charged and discharged to 1.5 V, then were decomposed. The electrode pellets were directly conducted on Raman.

### XPS

XPS measurement was performed using Kratos AXIS Ultra^DLD^ spectrometer applying a monochromated Al Ka X-ray source (1486.71 eV photons) and a dwelling time of 100 ms. The binding energy of the spectra was calibrated against *C* 1 s peak at 285 eV. The XPS spectra were mathematically fitted using XPSPEAK41 software. Core peaks were analysed using a Linear-type background. For XPS mesurement of pristine sample, the as-prepared Na_2_Ti_0.94_Cr_0.06_O_2.97_ powder was sticked on the sample hold of the XPS spectrometer. For the XPS mesurements of charge-discharge electrodes, the charged and discharged electrodes were stripped from the Al foils and dried in glove box and were sticked on the sample hold of the XPS spectrometer in air, and were performed XPS measurements immediately to avoid exposing longly to air.

### Density Functional Calculations

Density Functional Theory (DFT) was used to optimize crystalline structures with the SIESTA code^37^. The PBE exchange-correlation functional and a DZP (double-ζ polarized) basis set were used^38^. Spin polarization was checked and found to be unimportant. A TZP (triple–ζ polarized) basis was tested and did not significantly change the results vs. DZP. The basis set for Na was optimized to reproduce the cohesive energies of Na. For other elements, the basis set was generated with the setting “PAO.EnergyShift = 0.002 Ry”. Geometries were optimized until forces on all atoms were below 0.02 eV/Å and pressure was below 0.1 GPa. A cutoff of 200 Ry was used for the Fourier expansion of the density. An electronic temperature of 1000 K was used to facilitate convergence. The Brillouin zone was sampled with a 5 × 3 × 3 grid of Monkhorst-Pack points^39^. The initial lattice parameters of the unit cell are a = 5.37 Å, b = 9.31 Å and c = 11.07 Å; *α* = 90.00°, *β* = 99.68° and *γ* = 90.00° for Na_16_Ti_8_O_24_, which is taken from the Material Project with ID of mp-752423^40^. The optimized Na_16_Ti_8_O_24_ shows lattice parameters about: *a* = 5.39 Å, *b* = 9.36 Å and *c* = 11.02 Å; *α* = 90.00°, *β* = 99.70° and *γ* = 90.00°, which are in good agreement with the initial ones. The trend of charges of each element on the number of Na atoms in the formula was analyzed qualitatively by using Mulliken charges.

## Electronic supplementary material

Supplementary

## References

[1] Tarascon J-M (2010). Is lithium the new gold. Nature Chem.

[2] Yabuuchi N, Kubota K, Dahbi M, Komaba S (2014). Research development on sodium-ion batteries. Chem. Rev..

[3] Dunn B, Kamath H, Tarascon J-M (2011). Electrical energy storage for the grid: a battery of choices. Science.

[4] Kim S, Seo D, Ma X, Ceder G, Kang K (2012). Electrode materials for rechargeable sodium-ion batteries: potential alternatives to current lithium-ion batteries. Adv. Energy Mater.

[5] Delmas C, Fouassier C, Hagenmuller P (1980). Structural classification and properties of the layered oxides. Physica B + C.

[6] Zhao J, Zhao L, Dimov N, Okada S, Nishida T (2013). Electrochemical and thermal properties of α-NaFeO_2_ cathode for Na-ion batteries. J. Electrochem. Soc..

[7] Yu CY (2015). NaCrO_2_ cathode for high-rate sodium-ion batteries. Energy Environ. Sci..

[8] Han MH, Gonzalo E, Casas-Cabanas M, Rojo T (2014). Structural evolution and electrochemistry of monoclinic NaNiO_2_ upon the first cycling process. J. Power Sources.

[9] Yabuuchi N (2012). P2-type Na_x_[Fe_1/2_Mn_1/2_]O_2_ made from earth-abundant elements for rechargeable Na batteries. Nat. Mater..

[10] Ma XH, Chen HL, Ceder G (2011). Electrochemical properties of monoclinic NaMnO_2_. J. Electrochem. Soc..

[11] Billaud J (2014). Beta-NaMnO_2_: a high-performance cathode for sodium-ion batteries. J. Am. Chem. Soc.

[12] Billaud J (2014). Na0.67Mn_1−x_Mg_x_O_2_ (0 ≤ × ≤ 0.2): a high capacity cathode for sodium-ion batteries. Energy Environ. Sci..

[13] Lee E (2014). Layered P2/O3 intergrowth cathode: toward high power Na-ion batteries. Adv. Energy Mater.

[14] Guo SH (2015). A layered P2- and O3-type composite as a high-energy cathode for rechargeable sodium-ion batteries. Angew. Chem. Int. Ed..

[15] Jiang Y (2015). Nanoconfined carbon-coated Na_3_V_2_(PO_4_)_3_ particles in mesoporous carbon enabling ultralong cycle life for sodium-ion batteries. Adv. Energy Mater.

[16] Kim J (2015). Unexpected discovery of low-cost maricite NaFePO_4_ as a high-performance electrode for Na-ion batteries. Energy Environ. Sci..

[17] Ha K (2013). Na_4−α_M_2+α/2_(P_2_O_7_)_2_ (2/3 ≤ α ≤ 7/8, M = Fe, Fe_0.5_Mn_0.5_, Mn): A promising sodium ion cathode for Na-ion batteries. Adv. Energy Mater.

[18] Hill WA, Moon AR (1985). Alkali oxide rich sodium titanates. J. Am. Ceram. Soc..

[19] Bamberger CE, Begun GM (1987). Sodium titanates: stoichiometry and Raman spectra. J. Am. Ceram. Soc..

[20] Thackeray MM (2007). Li_2_MnO_3_-stabilized LiMO_2_ (M = Mn, Ni, Co) electrodes for lithium- ion batteries. J. Mater. Chem..

[21] Doeff MM, Hu Y, McLarnon F, Kostecki R (2003). Effect of surface carbon structure on the electrochemical performance of LiFePO_4_. Electrochem.Solid-State Lett.

[22] Rajarathinam S, Mitra S, Petla RK (2013). Li_2_MnO_3_ rich-LiMn_0.33_Co_0.33_Ni_0.33_O_2_ integrated nano-composites as high energy density lithium-ion battery cathode materials. Electrochim. Acta.

[23] Yabuuchi N, Yoshii K, Myung S-T, Nakai I, Komaba S (2011). Detailed studies of a high- capacity electrode material for rechargeable batteries, Li_2_MnO_3_-LiCo_1/3_Ni_1/3_Mn_1/3_O_2_. J. Am. Chem. Soc..

[24] Xu Y (2004). Electronic structure and electrical conductivity of undoped LiFePO_4_. Electrochem. Solid-State Lett.

[25] Rozier P (2015). Anionic redox chemistry in Na-rich Na_2_Ru_1-y_Sn_y_O_3_ positive electrode material for Na-ion batteries. Electrochem. Commun..

[26] Ruther RE (2015). Raman microscopy of lithium-manganese-rich transition metal oxide cathodes. J. Electrochem. Soc..

[27] Shchukarev A, Boily J-F, Felmy AR (2007). XPS of fast-frozen hematite colloids in NaCl aqueous solutions: I. evidence for the formation of multiple layers of hydrated sodium and chloride ions induced by the{001}basal plane. J. Phys. Chem. C.

[28] Liu C, Tang X, Mo C, Qiang Z (2008). Characterization and activity of visible-light-driven TiO_2_ Photocatalyst codoped with nitrogen and cerium. J. Solid State Chem..

[29] Wang YS (2015). Ti-substituted tunnel-type Na_0.44_MnO_2_ oxide as a negative electrode for aqueous sodium-ion batteries. Nat. Commun..

[30] Dupin JC, Gonbeau D, Vinatier P, Levasseur A (2000). Systematic XPS studies of metal oxides, hydroxides and peroxides. Phys. Chem. Chem. Phys..

[31] Dahéron L (2008). Electron transfer mechanisms upon lithium deintercalation from LiCoO_2_ to CoO_2_ investigated by XPS. Chem. Mater..

[32] Okonkwo IA (2012). Oxidation states of molybdenum in oxide films formed in sulphuric acid and sodium hydroxide. Thin Solid Films.

[33] Dedryvere R (2010). Electrode/electrolyte interface reactivity in high-voltage spinel LiMn_1.6_ Ni_0.4_O_4_/Li_4_Ti_5_O_12_ lithium-ion battery. J. Phys. Chem. C.

[34] Xu M, Lu D, Garsuch A, Lucht BL (2012). Improved performance of LiNi_0.5_Mn_1.5_O_4_ cathodes with electrolytes containing dimethylmethylphosphonate (DMMP). J. Electrochem. Soc..

[35] Sathiya M (2013). Reversible anionic redox chemistry in high-capacity layered-oxide electrodes. Nat. Mater..

[36] Han MM, Gonzalo E, Singh G, Rojo T (2015). A comprehensive review of sodium layered oxides: powerful cathodes for Na-ion batteries. Energy Environ. Sci..

[37] Kohn W, Sham LJ (1965). Self-consistent equations including exchange and correlatioin effects. Phys. Rev.

[38] Perdew JP, Burke K, Ernzerhof M (1996). Generalized gradient approximation made simple. Phys. Rev. Lett..

[39] Monkhorst HJ, Pack JD (1976). Special popints for Brillouin-zone integrations. Phys. Rev. B.

[40] *Materials Project Network* https://www.materialsproject.org/materials/mp-752423/.

