# Supplementary material for: Na-rich layered Na2Ti1−xCrxO3−x/2 (x = 0, 0.06): Na-ion battery cathode materials with high capacity and long cycle life
Source: Sci Rep. 2017 Mar 23;7:373. doi: 10.1038/s41598-017-00346-x (PMC5428299; doi:10.1038/s41598-017-00346-x)
Supplement: Supplementary file 1 — Supplementary [file 41598_2017_346_MOESM1_ESM.docx]

Supplementary Information

**Na-rich layered Na_2_Ti_1-x_Cr_x_O_3-x/2_ (x=0, 0.06): Na-ion battery cathode materials with high capacity and long cycle life**

Shufeng Song^1^, Masashi Kotobuki^2^, Yingqian Chen^2^, Sergei Manzhos^2^, Chaohe Xu^1^, Ning Hu^1,3^ & Li Lu^2,4^

^1^College of Aerospace Engineering, Chongqing University, Chongqing 400044, P.R. China. ^2^Materials Science Group, Department of Mechanical Engineering, National University of Singapore, Singapore 117575. ^3^The State Key Laboratory of Mechanical Transmissions, Chongqing University, Chongqing 400044, P.R. China. ^4^National University of Singapore Suzhou Research Institute, Suzhou, P.R. China. Correspondence and requests for materials should be addressed to S.F.S. (email: [sfsong@cqu.edu.cn](mailto:sfsong@cqu.edu.cn)) or N.H. (email: [ninghu@cqu.edu.cn](mailto:ninghu@cqu.edu.cn)) or L.L. (email: luli@nus.edu.sg)

| Sample | Element | Area | RSF | Na/Ti |
| --- | --- | --- | --- | --- |
| Pristine | Na | 46956.1 | 1.685 | 12.3 |
|  | Ti | 4537.8 | 2.001 |  |
| Charged | Na | 22092.0 | 1.685 | 6.95 |
|  | Ti | 3777.4 | 2.001 |  |
| Discharged | Na | 30276.1 | 1.685 | 9.34 |
|  | Ti | 3849.6 | 2.001 |  |

**Table S1.** Na/Ti ratio of Na_2_Ti_0.94_Cr_0.06_O_2.97_ during charge and discharge.
